# Prediction of clinical non-response to methotrexate treatment in juvenile idiopathic arthritis

**DOI:** 10.1186/1546-0096-9-S1-P105

**Published:** 2011-09-14

**Authors:** EH Pieter Van Dijkhuizen, Maja Bulatović, Marloes W Heijstek, Nico M Wulffraat, Saskia MF Pluijm, Robert de Jonge

**Affiliations:** 1 Department of Pediatric Immunology, University Medical Centre Utrecht, Wilhelmina Children’s Hospital, Netherlands; 2 Department of Public Health, Erasmus University Medical Center Rotterdam, Netherlands; 3 Department of Clinical Chemistry, Erasmus University Medical Center Rotterdam, Netherlands

## Background

Methotrexate (MTX) is an efficacious drug in juvenile idiopathic arthritis (JIA). If JIA patients are unresponsive to MTX, effective combination treatment with biologicals is required to prevent joint damage.

## Aim

To develop a prediction model to identify MTX non-responders according to the American College of Rheumatology 70 criteria during the first year of treatment.

## Methods

Data was collected on 183 JIA patients. Clinical variables and single nucleotide polymorphisms (SNPs) in genes involved in the mechanism of action of MTX were determined at baseline. Using multivariate backward logistic regression, these variables were used to construct a prediction model for MTX non-response, whose diagnostic accuracy was evaluated. The model was subsequently validated in a cohort of 104 JIA patients.

## Results

The prediction model included: erythrocyte sedimentation rate and SNPs in genes coding for methionine synthase reductase, multidrug resistance 1, multidrug resistance protein 1 and proton-coupled folate transporter. The area under the receiver operating characteristics curve (AUROC) was 0.73 (95%CI: 0.64-0.81). The prediction model was transformed into a total risk score (range 0 to 11). At a cut-off score of ≥3, sensitivity was 78%, specificity 49%, positive predictive value was 83% and negative predictive value 41%. In the validation cohort, the AUROC was 0.65 (95%CI: 0.54-0.77).

## Conclusion

The prediction model we developed and validated combines clinical and genetic variables to identify JIA patients not responding to MTX treatment. This model could assist clinicians in making individualized treatment decisions.

